# Stem-cell-triggered immunity safeguards cytokinin enriched plant shoot apexes from pathogen infection

**DOI:** 10.3389/fpls.2014.00588

**Published:** 2014-10-30

**Authors:** Muhammad Naseem, Mugdha Srivastava, Thomas Dandekar

**Affiliations:** Functional Genomics and Systems Biology Group, Department of Bioinformatics, Biocenter, University of Wuerzburg, WuerzburgGermany

**Keywords:** auxin, stem cell niche, FLS2 receptor, CLAVATA3, cytokinins

## Abstract

Intricate mechanisms discriminate between friends and foes in plants. Plant organs deploy overlapping and distinct protection strategies. Despite vulnerability to a plethora of pathogens, the growing tips of plants grow bacteria free. The shoot apical meristem (SAM) is among three stem cells niches, a self-renewable reservoir for the future organogenesis of leaf, stem, and flowers. How plants safeguard this high value growth target from infections was not known until now. Recent reports find the stem cell secreted 12-amino acid peptide CLV3p (CLAVATA3 peptide) is perceived by FLS2 (FLAGELLIN SENSING 2) receptor and activates the transcription of immunity and defense marker genes. No infection in the SAM of wild type plants and bacterial infection in *clv3* and *fls2* mutants illustrate this natural protection against infections. Cytokinins (CKs) are enriched in the SAM and regulate meristem activities by their involvement in stem cell signaling networks. Auxin mediates plant susceptibility to pathogen infections while CKs boost plant immunity. Here, in addition to the stem-cell-triggered immunity we also highlight a potential link between CK signaling and CLV3p mediated immune response in the SAM.

## INTRODUCTION

Plants are deprived of mechanical articulation in parts of their bodies. Unlike animals, which defend themselves from enemies through fight and flight, plants are comparatively more vulnerable to damages caused by biotic and abiotic stresses due to their sessile life style. Also, by default, they are programmed to replace lost body organs such as leaves and flowers on regular basis. The pluripotent stem cells, which are the sustainer of their lifelong activities, provide a constant supply of precursor cells to form differentiated tissues and body organs (Aichinger et al., 2012). In plants, the shoot apical meristem (SAM), the root apical meristem (RAM), and the vascular meristem are the custodians of stem cells. These stem cell niches maintain a specific signaling environment to stop them from entering into differentiation all at once yet keep a required number of undifferentiated stem cells through a process of self-renewal (Aichinger et al., 2012; Hwang et al., 2012). Being a custodian of the next generation of plants through seeds and flowers, the SAM constantly supplies cells to meet the programming and contingency requirements and is expected to safeguard its integrity from agents that can derail its genetic preprogramming (Aichinger et al., 2012; Hwang et al., 2012).

It is noteworthy to mention that phytoplasmal infection reprograms the meristem determination. Thus it changes tomato plant apex architecture through pathogen induced meristem derailment (Wei et al., 2013). Likewise, by deploying 2b-suppressor protein, Cucumber Mosaic Virus (CMV) inhibits anti-viral RNA silencing surveillance system and causes infection in the SAM (Sunpapao et al., 2009). Despite the pathogen guided interventions in the SAM stem cell niches, disease free plants can be generated from the SAM and this underscores the sterile nature of the shoot apex. The exact mechanisms how the SAM stem cell niches are naturally immune was not known until recently. In this perspective article, we focus on recent reports delineating the mechanism of stem-cell-triggered immunity in the SAM (Lee et al., 2011, 2012a). We also highlight skepticism voiced (Mueller et al., 2012; Segonzac et al., 2012) and discuss future prospects regarding peptide-mediated stem cell signaling in plant immunity.

## OVERVIEW ON ROBUST SAM SIGNALING NETWORKS

**FIGURE 1:**
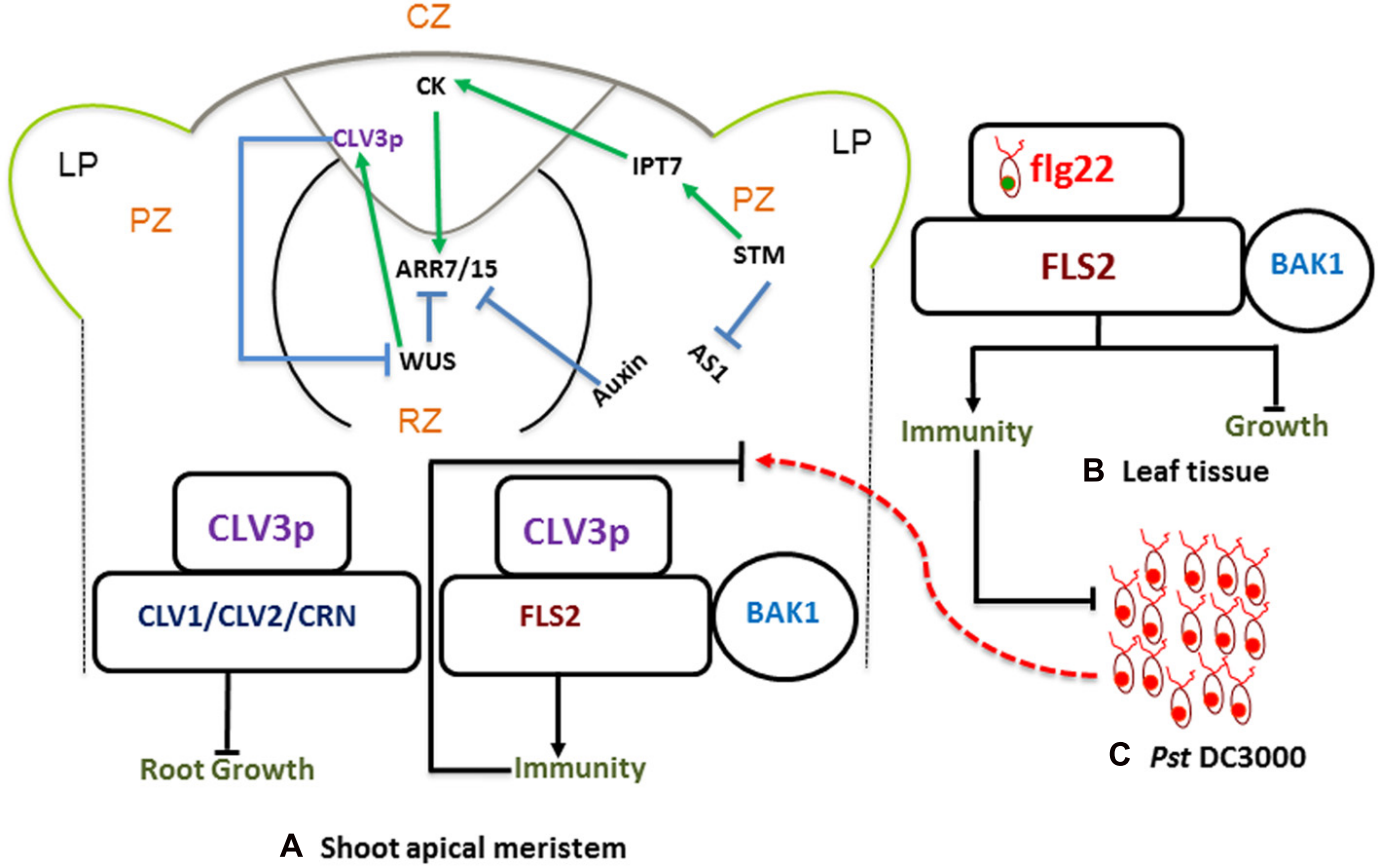
**Stem-Cell-Triggered Immunity.** Schematic diagram showing shoot apical meristem **(A)** protective and regulatory networks, driven by auxin and cytokinins (CKs). WUS and CLV3p mediated regulation play a pivotal role in maintaining a balance between proliferation and differentiation. CLV3p is perceived by the CLV-receptor complex and leads to maintenance of stem cell (left). There is an independent pathway where CLV3 also binds to FLS2 and activates innate immunity (right). This is analogous to typical bacterial flagellin perception mechanism, where flg22-FLS2 interaction triggers innate immunity in the leaf tissue **(B)** against infection with bacterial pathogens such as *Pst* DC3000 **(C)**.

The SAM is a dynamic structure of a hemispherical collection of identical appearing cells with a stable organization that maintain a balance between the self-renewal of stem cell population and conversion of meristematic cells into aerial organs such as shoot, leaves, and flowers (Perales and Reddy, 2012; Song et al., 2012). In *Arabidopsis* the SAM is comprised of three regions (**Figures 1A,B**), the central zone (CZ) is at the tip of the SAM and comprises the pluripotent stem cells. A collection of multipotent stem cells derived from the CZ constitutes the peripheral zone (PZ), from which the primordia of leaves and flowers come into existence (Aichinger et al., 2012). The rib meristem lies beneath the central and PZs, it turns into cells of the stem, as well as its vasculature. Plant hormone cytokinins (CKs) are believed to be the key signaling mediators in maintaining the integrity of the SAM stem cell niche (Hwang et al., 2012). In *Arabidopsis* enhanced plant CK responses stimulate meristem activities, whereas decreased CK signaling reduces meristem size (Nishimura et al., 2004; Bartrina et al., 2011). The expression of *SHOOT MERISTEMLESS* (*STM*) directly activates the transcription of the CK biosynthetic enzyme gene *ISOPENTENYLTRANSFERASE (IPT7).* Also, *stm-1* mutants are unable to initiate the SAM formation, suggesting that STM-mediated CK activation is important for the sustenance of the SAM (Aichinger et al., 2012). Besides the activation of CKs, STM also prevents the expression of the *ASYMMETRIC LEAVES1* (*AS1*) gene in the leaf primordium. Auxin represses the meristem promoting activities of CKs in the leaf primordium, while *STM* represses *AS1* in the meristem (Hwang et al., 2012; Perales and Reddy, 2012; **Figure 1A**).

In the CZ (**Figure 1A**), a pool of pluripotent stem cells is maintained by WUSHEL (WUS)/CLV3 mediated negative-feedback loop via CLAVATA1, 2 (CLV1/2) receptor signaling pathways (reviewed in: Matsubayashi, 2011; Aichinger et al., 2012; **Figure 1C**). CLV3p belongs to a family of 32 peptides called CLV3/EMBRYO SURROUNDING REGION peptide (CLEp), which is endogenously modified into mature (signaling) CLEp (Kondo et al., 2006; Gish and Clark, 2011) CKs induce the expression of *WUS* through their receptors ARABIDOPSIS HISTIDINE KINASE 2 and 4 (AHK2 and AHK4), but represses *CLV1* to avoid CLV3p-mediated *WUS* inhibition in the SAM (Hwang et al., 2012). In addition, members of CK type-A response regulators, ARABIDOPSIS RESPONSE REGULATORS 7 and 15 (ARR7 and ARR15), which activate CLV3p, are inhibited by AUXIN RESPONSE FACTOR 5 (ARF5), which in turn increases CK signaling and hence the induction of *WUS* (Aichinger et al., 2012; Perales and Reddy, 2012). These findings demonstrate that key nodes of stem cell signaling networks are under the regulatory control of auxin and CK signaling in the SAM.

## THE STEM CELL SIGNALING IMMUNITY MODEL AND CKs CROSSTALK

The importance of the CVL3/WUS-mediated module in the SAM, and the fact that mature 12-amino acid (aa) CLV3(peptide) p controls the maintenance of the SAM through CLV1 and CLV2 receptor complex (Hwang et al., 2012; Song et al., 2012) have already been well established. Besides these and similar receptors important for the development of SAM, CLV3p has been shown to interact with the well-known innate immune receptor FLS2. Analogous to flg22 (minimal 22-amino acid flagellin peptide, pathogen associated molecular pattern (PAMP): detailed in Sun et al., 2013), CLV3p binds to FLS2 and activates MITOGEN-ACTIVATING PROTEIN KINASE (MAPK) activities and induces the expression of PAMP-Triggered-Immunity (PTI) marker genes in *Arabidopsis* (Jones and Dangl, 2006; Lee et al., 2011). Unlike the flg22-FLS2 mediated immunity, which is coupled with growth inhibition of seedlings, the CLV3p-FLS2 activation results only in an immune response without growth inhibition (Lee et al., 2012a). Premature and precursor CLV3 peptides can be modified endogenously into various forms, such as 13aa-CLV3p and 12aa-CLV3p. However, FLS2 is shown to be specifically sensitive to 12-aa CLV3p (Lee et al., 2011, 2012b). These results suggest that there is a selective specificity for 12aa-CLV3p and that the FLS2 receptor seems to be blind to other variants of the same peptide.

These findings on stem-cell-triggered immunity were further complemented by genetic approaches (Lee et al., 2011). Accordingly, *fls2* mutant plants failed to show immune gene expression as well as MAPK activation by both CLV3p and flg22. However, the elongation factor EF-Tu (EFR) receptor mutant *efr-1* showed normal immune response both to flg22 and CLV3p (Lee et al., 2012a). Moreover, *clv1* and *clv2* mutants that fail to perceive CLV3p, showed normal immune gene activation without root length reduction. This underscores that CLV3p binds to distinct receptors for mediating growth and immunity (**Figure 1**). Intriguingly, when the SAM of L*er* plants was inoculated with *Pseudomonas syringae* pv. tomato DC3000 (*Pst* DC3000), no bacteria were detected 3-days post pathogen inoculation. However, substantial bacterial growth was found in the SAM of *clv3* as well as *fls2* mutants. This evidence further augments the concept of CLV3p mediated stem-cell-triggered immunity in *Arabidopsis*. Despite the fact that this mutually exclusive evidence strongly indicates the importance of CLV3p and FLS2 mediated stem-cell-triggered immunity, there still is some skepticism about the binding specificity of FLS2 and CLV3p. The physiological relevance of the different infection assays, as well as the effective concentrations of CLV3p in immunity and growth are among the well-taken caveats voiced by various groups.

While attributing the CLV3p-FLS2 mediated stem-cell-triggered immunity to an inadvertent contamination by flagellin, Mueller et al. (2012) demonstrated that in comparison to 1 nM flg22, 100 μM CLV3p proved to be blind to FLS2 in eliciting immune responses in their devised assay. They elegantly demonstrated that CLV3p does not induce the expression of reporter *pFRK1:Luciferase* via the receptor FLS2. The binding between CLV3p and FLS2, unlike that of flg22, and FLS2, cannot be confirmed. Moreover, an independent study by Segonzac et al. (2012) claimed the inability of *Pst* DC3000 to cause infection in the SAM is by virtue of topological hindrance instead of active immune defense. Several of these apprehensions have already been addressed and the newly published data (Lee et al., 2012a,b) reinstate that the basic finding is still in place.

It is interesting to see that flg22 and CLV3p are quite different in sequence as well as in structure (Shinohara and Matsubayashi, 2013; Sun et al., 2013), yet both are recognized by FLS2 in triggering downstream immune signaling (Lee et al., 2011). In contrast, very subtle differences in various CLV3 peptides greatly affect their recognition by FLS2. There is a peculiar high binding specificity between 12-aa CLV3p and FLS2 in mediating stem cell trigged immunity in the SAM (Lee et al., 2012a). Interestingly, a truncated version of CLV3p, the 11-aa CLV3p with missing terminal histidine in the C-terminus of 12-aa CLV3p also could not show binding to FLS2 and associated immune response in the SAM (Lee et al., 2011). Furthermore, 13-aa CLV3p (addition of a histidine in the C-terminus of 12-aa CLV3p, which is the most active and native mature form) failed to show immune gene expression (Lee et al., 2012a,b). However, addition of a tyrosine in the N-terminus of the 12-aa CLV3p displayed an immune response similar to that of native 12-aa CLV3p (Lee et al., 2012a). These results underscore the importance of the C-terminal residues in the native 12-aa CLV3p signaling peptide in invoking immune responses in the SAM. In addition, differences in binding to active sites of FLS2 by CLV3p and flg22 seem to invoke differential binding affinities on the same receptor (Lee et al., 2011). Moreover, physiologically both these ligands require a different pH to show their peak activities, while interacting with FLS2 (Lee et al., 2012b). Therefore, we can infer that the nature of the binding behaviors of flg22 and that of CLV3p to receptor FLS2 are different. Whereas the crystal structure of the binding complex between flg22 and FLS2-BRASSINOSTEROID INSENSITIVE 1-ASSOCIATED KINASE 1 (BAK1) has already been solved (Sun et al., 2013), the preliminary observations concerning CLV3p and FLS2 require detailed structural elucidation. Only then we would be able to understand fully the binding dynamics of such two structurally divergent ligands for the well-known plant immune receptor FLS2.

It is worth noting that biosynthesis and metabolism of CKs regulate meristematic activities in plant apexes. For instance, the *LONELY GUY* (*LOG*) gene encodes an enzyme that converts inactive CKs into free active bases and is specifically expressed at the top of apical meristem (Kurakawa et al., 2007). These and similar studies established the notion that CKs are enriched in the SAM and regulate stem cells homeostasis in these niches (Hwang et al., 2012). Higher CK levels and signaling have been shown to activate defense against infection with *Pst* DC3000 and *Hyaloperonospora arabidopsidis* (Argueso et al., 2012; Naseem et al., 2014), whereas auxin responses (Navarro et al., 2006; Naseem et al., 2012) promote susceptibility at the whole plant level. Generally, CLV signaling pathway in the SAM is partially regulated by plant hormone CKs. Existence of CLV3p is not only important for the regulation of meristem activities but also proved indispensable for the FLS2 mediated immunity, as *clv3* mutants are compromised in such immune responses (Lee et al., 2011; Aichinger et al., 2012). It has been investigated that CKs signaling regulate CLV3p through multiple loops. For instance; CK perception through AHK2 and AHK4 activates ARR7 and ARR15, and these type-A ARRs promote CLV3p signaling (Hwang et al., 2012; Perales and Reddy, 2012). Likewise, CK perception also activates WUS, which positively regulates CLV3p. WUS is inhibited by CLV3p, through the CLV1 pathway in the SAM as a negative feedback loop, (Hwang et al., 2012). These findings point to fact that CK signaling has a regulatory impact on the signaling of CLV3p and that immune responses executed through CLV3p-FLS2 pathway may be modulated by CK actions in the SAM. However, neither the direct effect of CKs on CLV3-FLS2 immune signaling, nor its link to the Salicylic acid–Jasmonic Acid (SA–JA) backbone of plant immunity has been investigated yet in the SAM. Nevertheless, transcriptional regulation by WUS revealed strong repression of JA response factor *JAZ5*, regulation of auxin responses, and the activation of CK signaling (Busch et al., 2010). Regulation of auxin has direct impact on the jasmonate, as well as salicylate immune pathways in plants. Repression of JAZs might have an inducing impact on jasmonate immune responses whereas WUS mediated CK signaling may positively influence plant immunity by regulating SA mediated defenses. Therefore, a detailed investigation focusing on the broad hormonal interplay regulating immune responses in the SAM is needed.

## AN OUTLOOK ON STEM-CELL-TRIGGERED IMMUNITY

An extraordinary mechanism of bacterial cleansing with immune defense is deployed by stem cells in the SAM (Lee et al., 2012b). This type of immunity has great similarities to that of flg22-FLS2-triggered innate immune response in plants, such as the dimerization of co-receptor BAK1 and downstream signaling events (Asai et al., 2002; Boller and Felix, 2009). It is worth mentioning that flg22-FLS2-based immunity is transient by nature and has often been viewed as a small increment of the total effective immunity executed through plant immune systems (Jones and Dangl, 2006). On the contrary, CLV3p-FLS2 mediated immunity seems to result in complete removal of *Pst* DC3000 in the SAM, whereas *fls2* and *clv3* mutants showed some susceptibility to bacterial infection (Lee et al., 2012a). However, the multiplication of *Pst* DC3000 in susceptible SAM of *fls2* or *clv3* mutant plants is lower than in a typical compatible infection in *Arabidopsis* (Segonzac et al., 2012). This sluggish bacterial growth in the SAM may be due to the compactness of the meristematic tissues leading to reduced space for the optimal bacterial multiplication, as compared with apoplastic compartments in plants. Alternatively, there might exist CLV3p-FLS2 independent protection mechanisms in susceptible SAM. Owing to the scarcity of detailed literature on stem-cell-triggered immunity in plants, the underlying mechanism delineating how *Pst* DC3000 fails to dampen CLV3p-FLS2 mediated immunity in the SAM remains to be determined. We suggest the following hypotheses to address the lack of effectiveness of *Pst* DC3000 effectors in breaching CLV3p-FLS2 mediated immunity in the SAM:

(i) Unlike apoplastic fluid, which is analogous to *hrp* inducing medium (Zwiesler-Vollick et al., 2002; Rico and Preston, 2008), the SAM microenvironment may not be favorable for the expression of bacterial genes implicated in Type III secretion system (TSS), responsible for the delivery of bacterial effectors into the plant cell; (ii) The distinction between PAMPs and effectors sometime cannot strictly be maintained (Thomma et al., 2011), therefore CLV3p may also be recognized by cellular receptors (*R*-genes) analogous to those of TSS delivered bacterial effectors and may execute Effector-Triggered-Immunity (ETI), which is higher in magnitude than PTI (Jones and Dangl, 2006); (iii) Whereas hypersensitive response (HR) and systemic resistance through meristems have already been demonstrated against infection of *Phytophthora infestans* (Orłowska et al., 2012), the impetus of such responses might be strong enough to prevent bacterial infection in the SAM as compared to other parts of the plant. These different hypotheses merit detailed experimental clarification in the future.

Once fully explored, the introduction of CLV3p mediated immunity at novel locations in plant could be a smart solution to improve plant protection against pathogens, thus avoiding too high growth cuts. However, to engineer this pathway in vegetative plant parts such as leaves, root, and stem, developmental complications of spatial and temporal nature may arise. To counter such problems, context dependent inducible gene expression systems would be a solution (Rushton et al., 2002; Großkinsky et al., 2011). Genetically modified plants expressing the CLV3p peptide under the control of an elicitor or a pathogen inducible promoter will drive transgene expression only at the onset of pathogen infection. Nevertheless, unforeseen complexities such as gene silencing and non-specific traits in transgenic plants remain valid concerns.

## Conflict of Interest Statement

The authors declare that the research was conducted in the absence of any commercial or financial relationships that could be construed as a potential conflict of interest.

## References

[B1] Aichinger E. Kornet N. Friedrich T. Laux T. (2012). Plant stem cell niches. *Annu. Rev. Plant Biol.* 63 615–636. 10.1146/annurev-arplant-042811-105555 22404469

[B2] Argueso C. T. Ferreira F. J. Epple P. To J. P. C. Hutchison C. E. Schaller G. E. (2012). Two-component elements mediate interactions between cytokinin and salicylic acid in plant immunity. *PLoS Genet.* 8:e1002448. 10.1371/journal.pgen.1002448 PMC3266875 22291601

[B3] Asai T. Tena G. Plonikova J. Willmann M. Chiu W.-L. Gomez-Gomez L. (2002). MAP kinase signaling cascade in *Arabidopsis* innate immunity. *Nature* 415 977–983. 10.1038/415977a 11875555

[B4] Bartrina I. Otto E. Strnad M. Werner T. Schmülling T. (2011). Cytokinin regulates the activity of reproductive meristems, flower organ size, ovule formation, and thus seed yield in *Arabidopsis thaliana*. *Plant Cell* 23 69–80. 10.1105/tpc.110.079079 21224426 PMC3051259

[B5] Boller T. Felix G. (2009). A renaissance of elicitors: perception of microbe-associated molecular patterns and danger signals by pattern-recognition receptors. *Annu. Rev. Plant Biol.* 60 379–406. 10.1146/annurev.arplant.57.032905.105346 19400727

[B6] Busch W. Miotk A. Ariel F. D. Zhao Z. Forner J. Daum G. (2010). Transcriptional control of a plant stem cell niche. *Dev. Cell* 18 841–853. 10.1016/j.devcel.2010.03.012 20493817

[B7] Gish L. A. Clark S. E. (2011). The RLK/Pelle family of kinases. *Plant J.* 66 117–127. 10.1111/j.1365-313X.2011.04518.x 21443627 PMC4657737

[B8] Großkinsky D. K. Naseem M. Abdelmohsen U. R. Plickert N. Engelke T. Griebel T. (2011). Cytokinins mediate resistance against *Pseudomonas syringae* in tobacco through increased antimicrobial phytoalexin synthesis independent of salicylic acid signaling. *Plant Physiol.* 157 815–830. 10.1104/pp.111.182931 21813654 PMC3192561

[B9] Hwang I. Sheen J. Müller B. (2012). Cytokinin signaling networks. *Annu. Rev. Plant Biol.* 63 353–380. 10.1146/annurev-arplant-042811-105503 22554243

[B10] Jones J. D. Dangl J. L. (2006). The plant immune system. *Nature* 444 323–329. 10.1038/nature05286 17108957

[B11] Kondo T. Sawa S. Kinoshita A. Mizuno S. Kakimoto T. Fukuda H. (2006). A plant peptide encoded by CLV3 identified by in situ MALDI-TOF MS analysis. *Science* 313 845–848. 10.1126/science.1128439 16902141

[B12] Kurakawa T. Ueda N. Maekawa M. Kobayashi K. Kojima M. Nagato Y. (2007). Direct control of shoot meristem activity by a cytokinin-activating enzyme. *Nature* 445 652–655. 10.1038/nature05504 17287810

[B13] Lee H. Khatri A. Plotnikov J. M. Zhang X. C. Sheen J. (2012a). Complexity in differential peptide-receptor signaling: response to Segonzac et al. and Mueller et al. commentaries. *Plant Cell* 24 3177–3185. 10.1105/tpc.112.099259 22923676 PMC3462623

[B14] Lee H. Chah O. K. Plotnikov J. Sheen J. (2012b). Stem cell signaling in immunity and development. *Cold Spring Harb. Symp. Quant. Biol.* 77 75–81. 10.1101/sqb.2012.77.014837 23174766

[B15] Lee H. Chah O. K. Sheen J. (2011). Stem-cell-triggered immunity through CLV3p-FLS2 signalling. *Nature* 473 376–379. 10.1038/nature09958 21499263 PMC3098311

[B16] Matsubayashi Y. (2011). Small post-translationally modified peptide signals in *Arabidopsis*. *Arabidopsis Book* 9:e0150. 10.1199/tab.0150 PMC3268502 22303274

[B17] Mueller K. Chinchilla D. Albert M. Jehle A. K. Kalbacher H. Boller T. (2012). Contamination risks in work with synthetic peptides: flg22 as an example of a pirate in commercial peptide preparations. *Plant Cell* 24 3193–3197. 10.1105/tpc.111.093815 22923674 PMC3462625

[B18] Naseem M. Philippi N. Hussain A. Wangorsch G. Ahmed N. Dandekar T. (2012). Integrated systems view on networking by hormones in *Arabidopsis* immunity reveals multiple crosstalk for cytokinin. *Plant Cell* 5 1793–1814. 10.1105/tpc.112.098335 22643121 PMC3442570

[B19] Naseem M. Wölfling M. Dandekar T. (2014). Cytokinins for immunity beyond growth, galls and green islands. *Trends Plant Sci.* 19 481–484. 10.1016/j.tplants.2014.04.001 24794463

[B20] Navarro L. Dunoyer P. Jay F. Arnold B. Dharmasiri N. Estelle M. (2006). A plant miRNA contributes to antibacterial resistance by repressing auxin signaling. *Science* 312 436–439. 10.1126/science.1126088 16627744

[B21] Nishimura C. Ohashi Y. Sato S. Kato T. Tabata S. Ueguchi C. (2004). Histidine kinase homologs that act as cytokinin receptors possess overlapping functions in the regulation of shoot and root growth in *Arabidopsis*. *Plant Cell* 16 1365–1377. 10.1105/tpc.021477 15155880 PMC490032

[B22] Orłowska E. Basile A. Kandzia I. Llorente B. Kirk H. G. Cvitanich C. (2012). Revealing the importance of meristems and roots for the development of hypersensitive responses and full foliar resistance to *Phytophthora infestans* in the resistant potato cultivar Sarpo Mira. *J. Exp. Bot.* 63 4765–4779. 10.1093/jxb/ers154 22844094 PMC3428001

[B23] Perales M. Reddy G. V. (2012). Stem cell maintenance in shoot apical meristem. *Curr. Opin. Plant Biol.* 15 10–16. 10.1016/j.pbi.2011.10.008 22079787

[B24] Rico A. Preston G. M. (2008). *Pseudomonas syringae* pv. tomato DC3000 uses constitutive and apoplast-induced nutrient assimilation pathways to catabolize nutrients that are abundant in the tomato apoplast. *Mol. Plant Microbe Interact.* 21 269–282. 10.1094/MPMI-21-2-0269 18184070

[B25] Rushton P. J. Reinstädler A. Lipka V. Lippok B. Somssich I. E. (2002). Synthetic plant promoters containing defined regulatory elements provide novel insights into pathogen- and wound-induced signaling. *Plant Cell* 14 749–762. 10.1105/tpc.010412 11971132 PMC150679

[B26] Segonzac C. Nimchuk Z. L. Beck M. Tarr P. T. Robatzek S. Meyerowitz E. M. (2012). The shoot apical meristem regulatory peptide CLV3 does not activate innate immunity. *Plant Cell* 24 3186–3192. 10.1105/tpc.111.091264 22923673 PMC3462624

[B27] Shinohara H. Matsubayashi Y. (2013). Chemical synthesis of *Arabidopsis* CLV3 glycopeptide reveals the impact of hydroxyproline arabinosylation on peptide conformation and activity. *Plant Cell Physiol.* 54 369–374. 10.1093/pcp/pcs174 23256149 PMC3589827

[B28] Song X. F. Yu D. L. Xu T. T. Ren S. C. Guo P. Liu C. M. (2012). Contributions of individual amino acid residues to the endogenous CLV3 function in shoot apical meristem maintenance in *Arabidopsis*. *Mol. Plant* 5 515–523. 10.1093/mp/ssr120 22259020

[B29] Sun Y. Li L. Macho A. P. Han Z. Hu Z. Zipfel C. (2013). Structural basis for flg22-induced activation of the *Arabidopsis* FLS2-BAK1 immune complex. *Science* 342 624–628. 10.1126/science.1243825 24114786

[B30] Sunpapao A. Nakai T. Dong F. Mochizuki T. Ohki S. T. (2009). The 2b protein of cucumber mosaic virus is essential for viral infection of the shoot apical meristem and for efficient invasion of leaf primordia in infected tobacco plants. *J. Gen. Virol.* 90 3015–3021. 10.1099/vir.0.013219-0 19675191

[B31] Thomma B. P. Nürnberger T. Joosten M. H. (2011). Of PAMPs and effectors: the blurred PTI-ETI dichotomy. *Plant Cell* 23 4–15. 10.1105/tpc.110.082602 21278123 PMC3051239

[B32] Wei W. Davis R. E. Nuss D. L. Zhao Y. (2013). Phytoplasmal infection derails genetically preprogrammed meristem fate and alters plant architecture. *Proc. Natl. Acad. Sci. U.S.A.* 110 19149–19154. 10.1073/pnas.1318489110 24191032 PMC3839765

[B33] Zwiesler-Vollick J. Plovanich-Jones A. E. Nomura K. Bandyopadhyay S. Joardar V. Kunkel B. N. (2002). Identification of novel hrp-regulated genes through functional genomic analysis of the *Pseudomonas syringae* pv. tomato DC3000 genome. *Mol. Microbiol.* 45 1207–1218. 10.1046/j.1365-2958.2002.02964.x 12207690

